# A new acquisition protocol for conducting studies with children: The science camp research experience

**DOI:** 10.1371/journal.pone.0289299

**Published:** 2023-08-09

**Authors:** Amparo V. Márquez-García, Sam M. Doesburg, Grace Iarocci, Justine R. Magnuson, Sylvain Moreno

**Affiliations:** 1 Department of Biomedical Physiology and Kinesiology, Simon Fraser University, Burnaby, Canada; 2 Department of Psychology, Simon Fraser University, Burnaby, Canada; 3 School of Health and Exercise Science, University of British Columbia Okanagan, Kelowna, BC, Canada; 4 Department of School of Interactive Arts & Technology, Simon Fraser University, Burnaby, Canada; University of Valencia, SPAIN

## Abstract

In the last 50 years, the study of brain development has brought major discoveries to education and medicine, changing the lives of millions of children and families. However, collecting behavioral and neurophysiological data from children has specific challenges, such as high rates of data loss and participant dropout. We have developed a science camp method to collect data from children using the benefits of positive peer interactions and interactive and engaging activities, to allow researchers to better collect data repeatedly and reliably from groups of children. A key advantage of this approach is that by increasing participant engagement, attention is also increased, thereby increasing data quality, reducing data loss, and lowering attrition rates. This protocol describes the step-by-step procedure for facilitation of a science camp, including behavioral, electrophysiological, and participatory engagement activities. As this method is robust but also flexible, we anticipate that it can also be applied to different demographics and research needs.

## 1. Introduction

Collecting data from children, with or without neurodevelopmental disabilities, has been notoriously challenging due to the characteristics of this population (e.g., lower attention spans, inter-variability, and emotional regulation difficulties). This has resulted in high rates of data loss, poor signal noise ratio and high participant dropout [1, 2]. Thus, these factors limit the amount of data that can be collected [3–5], resulting in many neurodevelopmental studies with small and unrepresentative samples and low statistical power, rendering the accuracy and reliability of study findings inadequate [6–9]. Evidently, there is a high current replicability crisis in neurodevelopmental field [6, 7]. Previous publications have presented recommendations for data collection of cognitive screening of children, some of which focus on specific pathologies like multiple sclerosis [10], autism [11], and pediatric traumatic brain injury [12]. Other publications are focused on recommendations for better electrophysiological data collection of children [13] and adults [14]. However, apart from recommendations for planning and conducting focus groups with children [15], there is a lack of real solutions in the extant literature to improve data collection in children. In this regard, there is an important need for a new acquisition protocol that provides a framework to collect high quality data with strong statistical power in studies with children. This methodological paper proposes a new acquisition protocol that addresses the main difficulties of data collection in children. It employs a system that allows researchers to collect a greater amount of high-quality data, utilizing strategies to engage participants and to achieve lower attrition rates and, thereby, more representative samples, as well as longer windows of attention and focus on tasks. This protocol is designed for researchers working with children to collect behavioural and/or neurophysiological data.

The standard version of this protocol has been used and validated with healthy and clinical populations of children aged 4 to 12 [16–20]. In this protocol, children are invited to participate in a one-day science camp. During their participation, they engage in a series of activities involving behavioural assessments, neurophysiological assessment, and various other ’fun’ activities. The participants are divided into groups, and each group performs one different activity at a time in a ‘circuit’, so that all groups have an opportunity to complete all activities by the end of the day. During the camp, the children have an opportunity to socialize throughout most of the activities. A great variety of behavioural, psychological, and neurophysiological assessments can be incorporated in this setting. One such neurophysiological assessment that can be easily applied is electroencephalography (EEG). Moreover, the ‘circuit’ of science camp protocol guides participants towards completing all necessary assessments while simultaneously providing a fun and rewarding interactive experience. Compared to traditional data collection methods, where one participant is evaluated at a time, the science camp method offers the opportunity to generate richer engagement between participants. Research has shown that positive peer interactions contribute to emotional regulation. Groups can provide positive interactions that influence the emotional disposition of children, encouraging greater participation in activities [21, 22].

In summary, this protocol aims to improve the quality and data collection process in developmental research, leading to a stronger research field with less contradictions and more reproduceable findings.

### 1.1 Development of the protocol

Challenges associated with conducting developmental research have been known for decades. Multiples strategies have been published from describing methods for running behavioural/psychometric tests [23], steps to administering EEG assessments [24–27], and different models of data preprocessing and analysis [28]. Studies have also described guidelines for administering EEG to children with autism [11], as well as common difficulties that arise with these assessments [29]. Each of these studies focused on improving one single task and did not prioritize the optimization of time and resources spent during data collection. Considerable progress has been made as the field has grown, and data collection has significantly improved over the years at the specific task level. Nevertheless, the main two problems of small samples and low data quality persist [6–9]. To address those two main issues, we developed a framework solution, the science camp protocol. Our goal was to collect behavioral and neurophysiological data from groups of children while making more efficient usage of time and resources (i.e., time, money, research assistants, participants) for data collection. What we proposed here is to implement a framework that results in improving those main issues simultaneously versus trying to improve a specific task.

Initially, we applied this protocol in studies collecting neurophysiological and behavioural data from large groups of children (i.e., 30 children in one day; [17, 19, 20, 30]). We also applied this protocol to investigate behavioural treatments/therapies efficacy in children including a pre-post evaluation [16–18, 31–33]. Lastly, we applied this protocol to evaluate behavioral differences between children with and without ASD [34–36]. Across this decade of studies, this method has been perfected to successfully collect usable data from approximately 80 children with and without neurodevelopmental disabilities per testing day [19, 20, 30]. Therefore, in this article, we will present guidelines for implementing a science camp protocol with children: from the experimental design to the additional activities necessary to implement a successful protocol.

### 1.2 Applications of the method

This science camp protocol has been used for behavioral and neurophysiological group data collection of children from 7 to 12 years old, with and without disabilities. This protocol can be used to address research questions such as investigating behavioural and neurophysiological processes during childhood (e.g., language, executive functions, etc.). In addition, this protocol could assist in several experimental design such as quasi-experimental and cross sectional. It can be used to study clinical or intervention trials in which researchers are investigating the outcomes of educational programs, treatment, or therapies. It could also be implemented in longitudinal design by repeating the protocol at different times. In terms of location, the protocol can be adapted in schools, hospitals and/or scientific laboratories, where many participants can be gathered [17, 19, 20], providing enough flexibility to be adapted to each group or setting’s needs.

### 1.3 Experimental design

The purpose of this article is to describe the implementation and execution of a science camp for behavioural and electrophysiological data collection in child populations. We recommend that this protocol be implemented by an experienced team in the collection of the selected type of data (i.e., specific behavioral tests) or the use of specific required equipment. We also assume a thorough understanding of the principles of behavioural and cognitive neuroscience. We focus on describing the unique opportunities and challenges presented in group data collection with children. While designing and performing these studies, it is of paramount importance to prioritize participant safety and comfort over research goals, as subjects who consent to participate without any expectation of direct benefit.

#### 1.3.1 Participants

Each participant should be evaluated regarding their suitability for the study prior to the day of data collection. Once the study’s guidelines have been determined and approved by the relevant ethics review board, we recommend adhering to the following steps involving communication with parents or guardians. As a general recommendation, the participants’ parents/caretakers should receive all the information about the testing procedure, including what to expect, how long it will take, etc. We recommend a parent meeting with the research team. It is crucial to ensure that the parent feels that their child will be able to tolerate the experiment without responding negatively to the procedures. Parents should be pre-consulted about the testing session’s date, time, and location. If parents and children agree to have their child participate, a simple to-do list should be provided to inform them of all the testing day recommendations. This to-do list is given by parents to children.

All study participants must be enrolled voluntarily, and caregivers and participants must provide informed written consent. In addition, participants must provide assent before each activity, they can decide not to participate in specific activities, and all subjects should be free to withdraw from the study or any activity involved at any time, including during the recording. We have different ways to reach out to our participants: a database and different communication tools: social media and daily newspapers add. See supplementary materials for an example of one of our advertisements used for recruitment (Fig 5 in S2 File).

#### 1.3.2 Planning

*1*.*3*.*2*.*1 Research team*. The large number of participants’ data typically collected using this method requires a large group of researchers. The group of assistants could be academics, clinicians, clinical staff, community professionals, and/or students. All should follow a proper experimental training to conduct the tasks of the protocol and understand how to work with children (including clinical child populations if necessary). All must have completed criminal background checks prior to data collection. Once the background checks have been approved, assistants should sign an agreement of collaboration and participate in the specific training sessions offered by the research group. The training should include an experimental aspect and a clinical aspect (if needed). The experimental aspect of the training should evaluate the previous knowledge of the research team members and create a plan to provide the knowledge they are missing.

After the training, all research team members should be prepared for the activity they were assigned. The group assigned to the administration of psychometric tests should complete the required training for each test (i.e., training varies greatly depending on the test). The groups assigned to a behavioral test that requires the use of a computer and electrophysiological measurement system, or any other equipment designed to make physiological recordings from participants should know how to connect and test all equipment; how to properly manipulate and clean them; how to run the task and record the data. In addition, they should know what to do if the participants refuse to participate in the activity (i.e., What room to direct the participants, what activities to offer instead, etc.).

*1*.*3*.*2*.*2 Piloting*. Several pilot testing sessions might be needed until all the assistants are capable of mastering the procedures and can do so in a specific timeframe. In the first pilot session, it is recommended that research team members take turns role-playing as participants, as children are not present at this stage. During the clinical part of the pilot stage, general and specific aspects of working with children should be included. Clear rules and expected behavior should always be stated from the beginning.

All research team members should know the characteristics of the group that they will work with and be prepared to act according to the participant’s needs, triggers, and level of functioning. One strategy to support this learning phase is to present case studies that represent different situations that teach assistants how to respond to each one. The pilot stage should be repeated as much as needed by the research team members.

*1*.*3*.*2*.*3 One day prior*. In order to ensure a successful group data collection, it is important to set up all data collection stations and run the experiments at least once on the day before the experiment, allowing the research group enough time to solve any last-minute issues that could arise. The research team members should receive clear instructions about who and how to contact for help if specific problems arise. Necessary checklists and information sheets should be provided for each research team member.

*1*.*3*.*2*.*4 Day of the data collection*. The day starts with an entry meeting in which the children and their parents are welcomed. All the parents sit down and receive a presentation (i.e., using pictures and videos) with the description of each activity and how the science camp works. Children are grouped within their age range. The research team provides children with their name tags and working materials such as t-shirts with different colors per group. The groups are gathered, and the parents and the children are introduced to their group leader (i.e., a research team member in charge of that group) and to their group mates. Time should be allotted to answer questions from parents and children. After introductions, children receive a card to help them collect one sticker for each activity completed. Research team members then lead their groups through the circuit of the science camp. Each group leader will stay the whole day with their group. It provides stability and reassurance for the children. Parents are welcome to stay in the registration room and participate in workshops, or they can run errands and come back before the exit meeting. At the end of the day, the protocol ends with an exit meeting. During this meeting, all groups of children, research team members and parents are reunited for a debriefing and answer any questions they may have. A recap of the event (i.e., showing pictures of the event to the parents) and prizes are provided. The research team will also inform the parents when they will be able to receive a report of the day. No individual report is shared with participant information but a global day description of what the children have done during the science camp protocol explaining the goal of the studies is encouraged to create a long-term relationship with the parents and their child.

#### 1.3.3 Data acquisition

**Fig 1:**
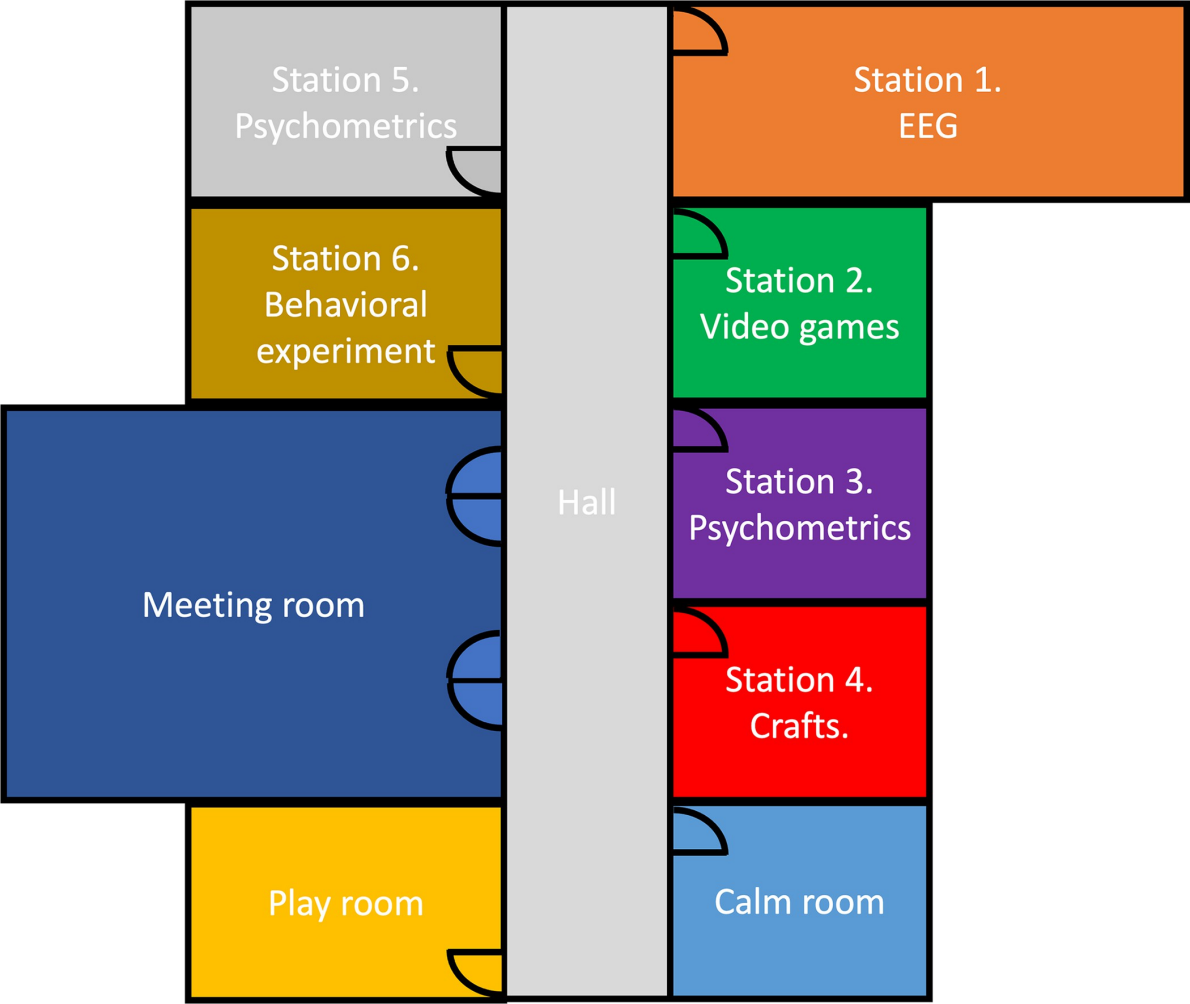
Science camp layout example.

**Fig 2:**
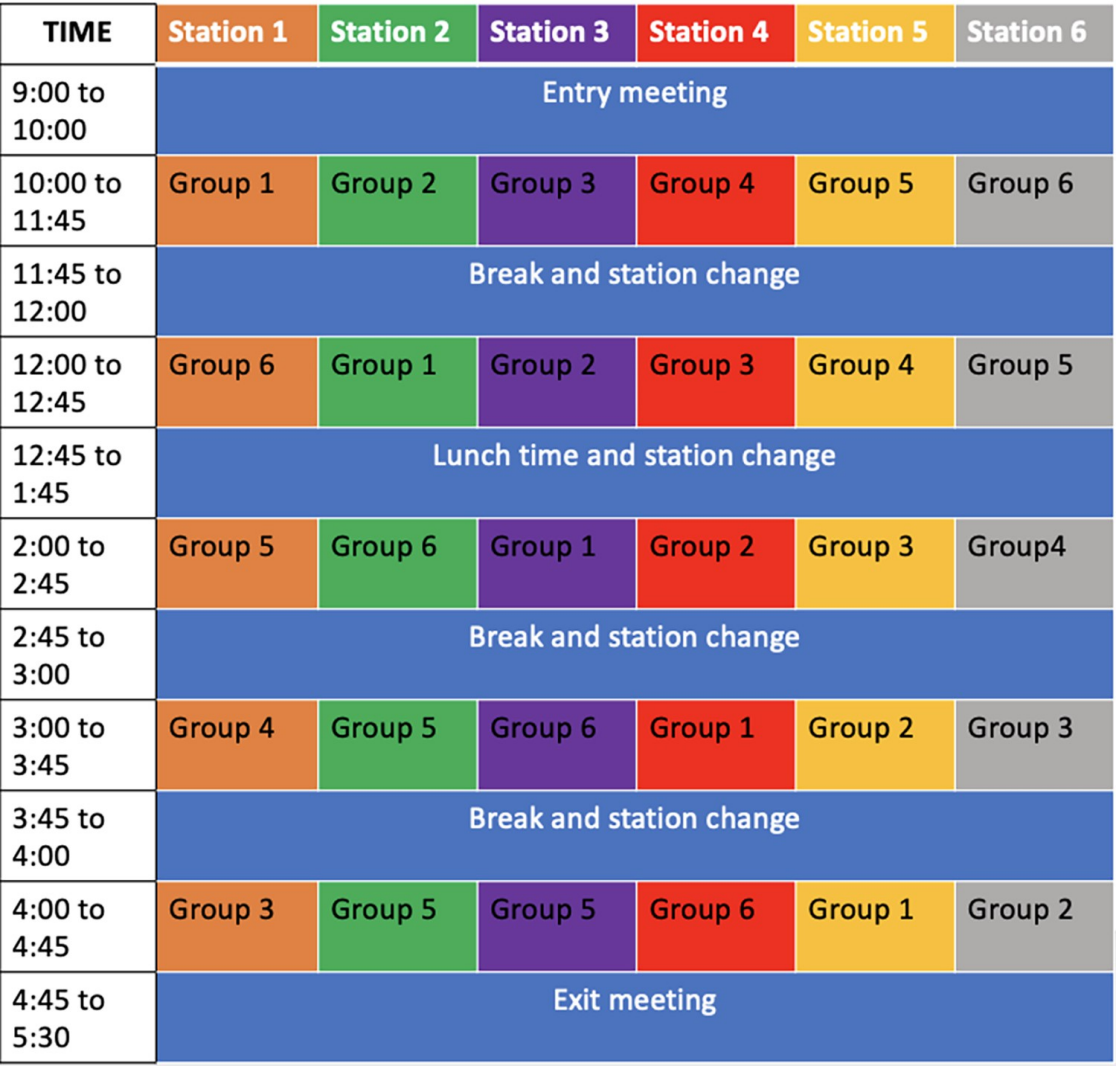
Science camp agenda showing the distribution of the groups and the activities.

To achieve a science camp protocol, we carefully planned a step-by-step one-day agenda. Different rooms are conditioned for different activities (see example in Fig 1). During the camp (approximately 9:00 a.m. to 5:00 p.m.), participants visit each of the stations where they will perform research-related and non-research related activities. Depending on the research question and the characteristics of the participants’ population, the activities, set up, and duration should be adapted. They will receive breaks in between each activity to be able to rest and engage with other participants (see example in Fig 2). In addition to the research and fun stations, it is necessary to prepare a room big enough to hold meetings with all the parents and participants. Fun station are two extra rooms for breaks, in these rooms there is always a research team member prepared to receive any participants who need a break. One of them is called the calm room; this room is always quiet. The second room is called the playroom; in this room, participants can take a break from the planned activities, and they have some extra options for fun activities to participate in (i.e., play board games, sing, draw or work on crafts).

In summary, the implementation of a science camp protocol will typically use at least three different types of stations. The first is for the behavioural evaluations; the second is the ‘fun’ stations, and the third is the electrophysiological station.

#### 1.3.4 Science camp behavioral assessment

**Fig 3:**
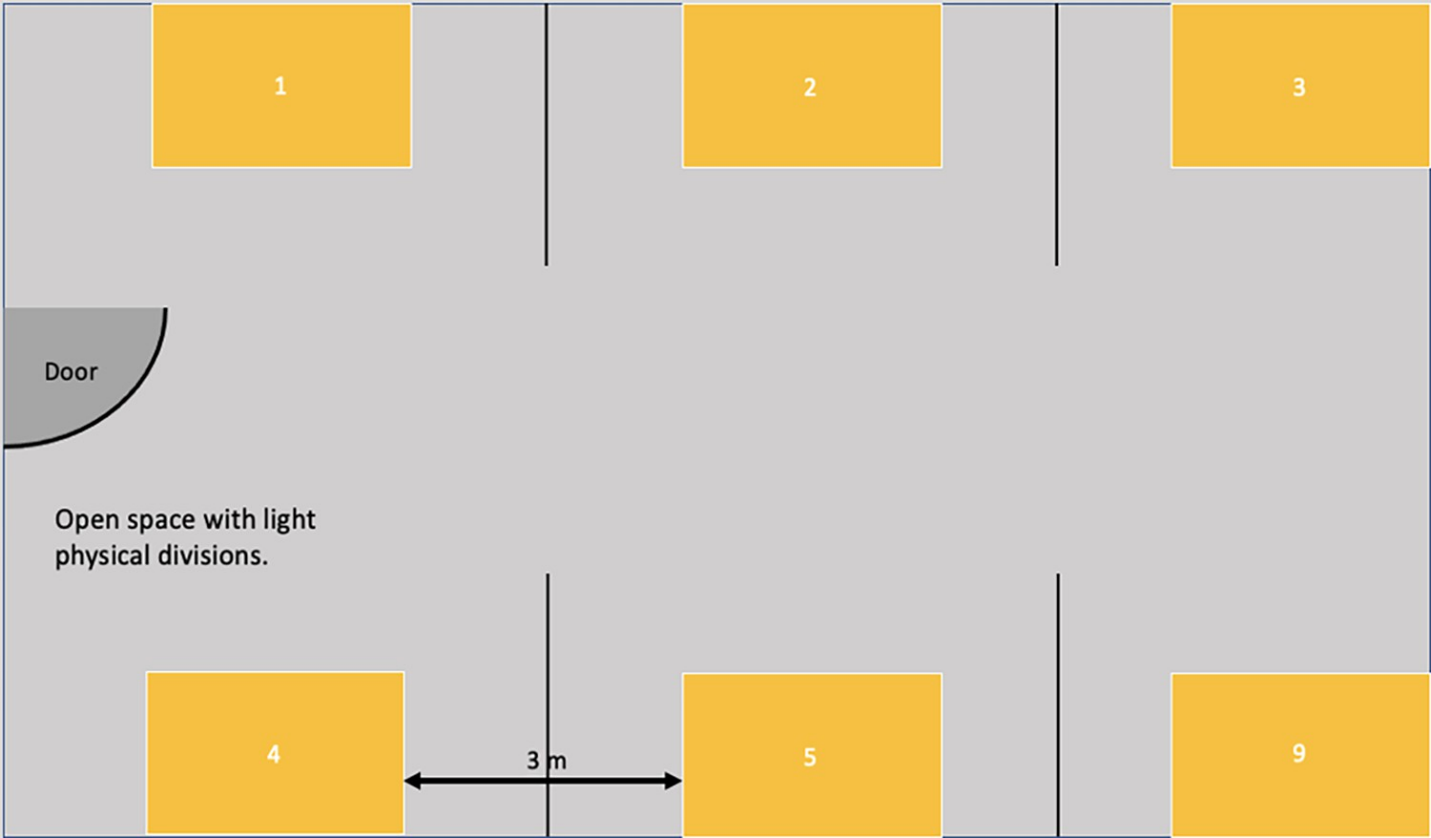
Example of layout in the room for psychometrics application.

The configuration of the rooms allows us to have the required privacy for psychometrics application or behavioural assessment simultaneously. It is important to have separate workstations with light divisions (i.e., Wood or plastic panels) to prevent children from interfering with each other’s activities in the same room (please see example in Fig 3).

#### 1.3.5 Science camp electrophysiology recordings

**Fig 4:**
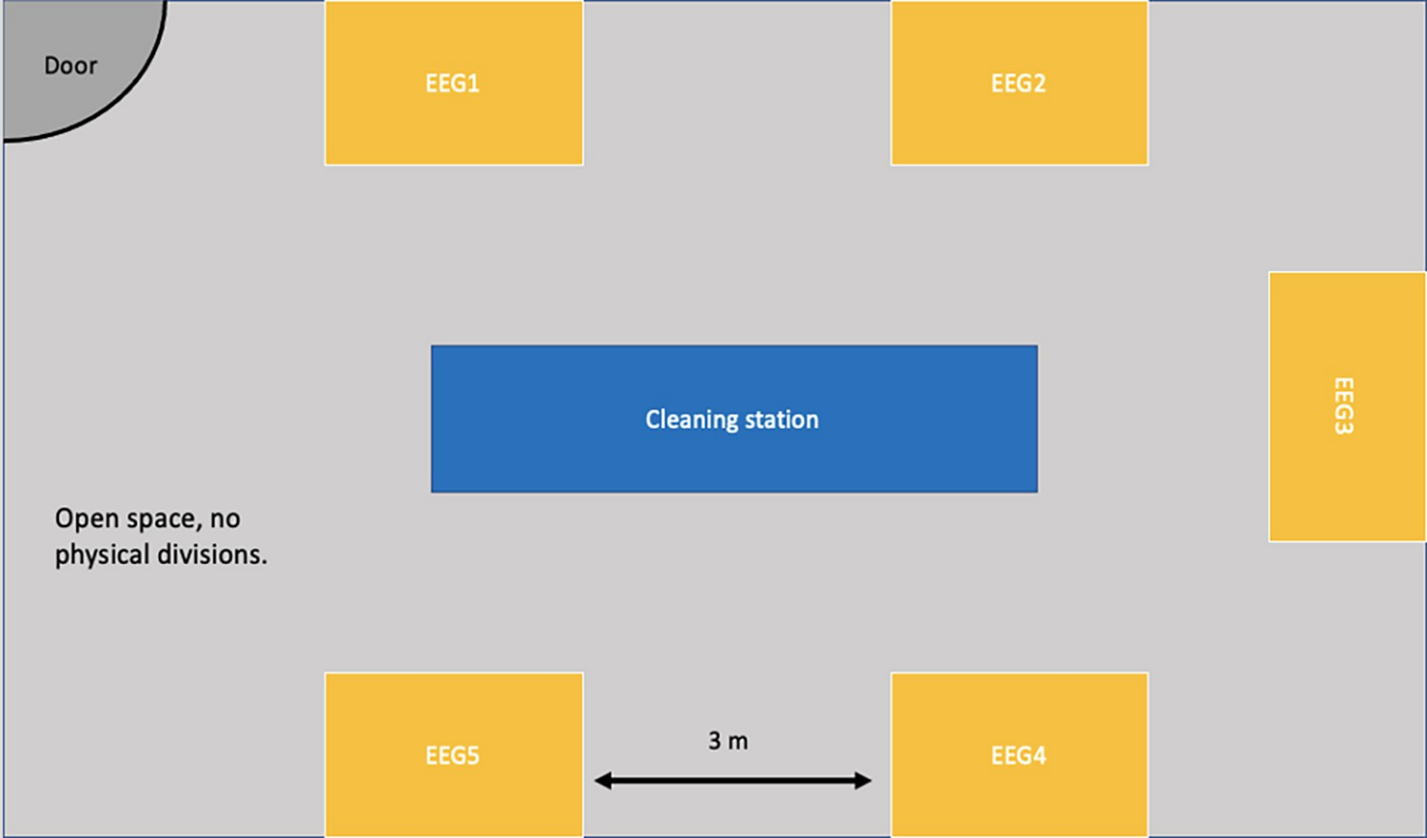
Layout of EEG recording room.

For the EEG station, our research group uses 5 EEG systems (i.e., the number of systems will depend on the space possibilities). The EEG station should be located in a room where EEG systems can be at least 3 meters apart and have one cleaning station in the center of the room (See Fig 4). Our data verification tests showed that 3 meters was enough to effectively minimize the signal contamination between systems. Having the cleaning station in the center of the room is convenient towards preparation of the stations between groups.

After the accent of the participants, they are guided to their station (one per participant). Two research assistants manage each station (i.e., two research assistants per station and participant). Within the first 20 minutes, the two research assistants explain the tasks’ instructions to each participant and prepare the settings (i.e., capping the participants and training them on the task).

In this setup, an essential factor is the orientation of the participants (i.e., not seeing each other), as this will limit the distraction and subsequent loss of data. During this time (the first 20 minutes), participants are also allowed to talk with the research assistants and move in their seats. For some participants, research assistants could also propose watching a short children’s movie to the participants.

The total length of the EEG recording depends on the research questions, task design and population. Previous tasks with children for our research group lasted up to 30 minutes, providing breaks between experimental blocks to allow them to stretch and move in their seats. Between groups, 15 minutes are necessary to clean and prepare the settings for the next group.

In total, 13 research assistants are needed in this station example. Each EEG system is operated by 2 research assistants. There is also one assistant coordinating the groups’ transitions, overseeing the overall data collection, and providing extra support if needed, another assistant ready to provide technical support and one more assistant working exclusively in the cleaning station preparing the supplies for all the groups.

#### 1.3.6 Science camp fun stations

These rooms can have a flexible configuration, but they should be safe for child play. These stations are where the participants have fun and interact with each other. For example, they can play board games, sing, draw or work on crafts. These stations can be adapted to the space and to the activities available and best suited to the participant populations. There is no need for a special configuration or separation between participants.

### 1.4 Expertise needed

The level of expertise required will depend on the specific role assigned during the data collection and the type of data collected. The research team is organized as a hierarchy according to the expertise needed. The researchers in charge of the project oversee and manage the study. In the leading roles of each activity (e.g., collection of behavioral data or electrophysiological recordings), it is necessary to have extensive experience in the application of these specific methodologies, tests, or tasks. In our published studies, students, and researchers with experience with the selected population of study occupied these roles. Each of the leaders will have to train and prepare a group of research team members in the specificities of their task (i.e., teach them how to collect electrophysiological data or how to apply a specific behavioral test). These research team members could be graduate or undergraduate students in areas related to the experiment. Lastly, the research team members in charge of transferring the children from one station to the other could be faculty, community professionals, parents, graduate students, and/or undergraduate students who have all completed criminal background checks and have completed the necessary training for the research group for this specific event. Experience working with the participant population is necessary.

### 1.5 Advantages

The science camp protocol has been created to improve the collection of behavioral and neurophysiological data from children with and without neurodevelopmental disabilities between the ages of 7 and 12 years old. The main advantages of this data collection setting are (i) possibility for a wide range of different tasks and activities (i.e., several tests), (ii) greater amount of data per participant (i.e., better signal noise ratio), (iii) higher participants’ performance and (iv) lower attrition rates, due to increased motivation from participants driven by fun activities and positive peer interactions, and (v) the flexibility of this protocol allows it to be adapted for use in multiple populations across various ages. In summary, the science camp protocol proposes a proven solution to improve the two main data collection issues of the child development research field: small participant samples and low data quality.

### 1.6 Limitations

A limitation of this protocol is the higher demand in planning, preparation, and management than what is required for more conventional protocols. Moreover, it necessitates a large research team. Additionally, the complexity of team training highlights the implementation difficulties of this protocol versus a classic protocol requiring the training of only one or two research team members. Finally, a large testing space is required. The facilities/testing environment needs to be large enough to hold the entire participant group at one time. We address possible problems and solutions on the section 4. Troubleshooting, Table 1.

**Table 1:** Example of possible problems and solutions during the science camp data collection day.

PROBLEM	SOLUTION
The child takes a break during the tasks	Encourage the child not to take breaks during the task, but if it is deemed necessary to prevent an outburst or some form of fatigue, invite the child to stay and remain quiet, or to join the playroom.
Child refuses to continue with the task or follow instructions properly	Remind them of the game and confirm that they no longer want to participate. If the answer is yes, invite the child to join a non-research activity (outside of the current testing room). It is unethical to keep a child at a station when they have made it clear either through verbal or nonverbal means that they do not wish to cooperate. If a child is being disruptive in the room, this could affect the quality and willingness of other children to participate. However, this may also be a sign that the child is no longer willing to participate.
Parents refuse a given task	Skip the task and send the participant to a fun activity, playroom, or calm room instead.
Child is afraid of the EEG caps or electrode gel	Make sure the child is able to squeeze the syringe and place some gel on their hands so they can understand for themselves that the gel is not dangerous (burning, glue-like, etc.). Also allow the participant to watch the caps being placed on the heads of other children or the experimenter to alleviate concerns.
The child begins to talk or move around	Psychometrics: make the pertinent annotations on the protocol and if necessary, exclude that section from the test analysis. EEG: note the time on the EEG recording and remove these trials from the data analysis. Also try to design a task that is engaging and short, if possible [37].
Problem with EEG equipment (e.g. Wi-Fi or Bluetooth connectivity issues, communication between task presentation and EEG acquisition computers)	To minimize connectivity issues, consider setting up equipment one day in advance and ensuring all the experiments run without any technical problems. In the case of any problems with equipment, we usually refer to the website of the manufacturer.
Participants are following the task protocol differently from each other	Limit the number of research assistants used in the research tasks and provide a script so that those who are explaining the task instructions to the participants are providing the same or very similar directions.
Technical difficulty in any station	Send participant to any of the other stations.

## 2. Materials

The following section provides a detailed illustration of our method providing a concrete example of the broader approach detailed above.

Informed assent from children and informed consent from parents and legal guardians must be obtained. The protocol must be in compliance with appropriate national laws and institutional regulatory board guidelines, and an appropriate ethics review board must approve the protocol for the sessions.

### Equipment

#### Psychometrics applications and cognitive tests

Complete psychometric battery and cognitive tests including the specific materials required for each one (i.e., parents’ questionnaires, application forms, white paper, colors, pencils, chronometer, etc.). The number of supplies should be enough to record each member of each subgroup of participants.

### The electrophysiological recording requires

- EEG equipment: the number of EEG systems should be enough to record each member of each subgroup of participants,
- cleaning supplies and recording supplies,
- one computer per EEG system with task presentation software,
- one computer per EEG system for data acquisition,
- data analysis software: MATLAB or other similar software is required to analyse the data,
- toys/games: a variety of toys are used to help participants develop rapport with the researchers while they wait for the equipment to be set up. Electric toys are not recommended, instead, sensory toys are recommended due to self-regulatory effects.

### Fun stations (examples)

- materials to create crafts (i.e., playdough, paper, watercolor paint, pipe cleaners, craft sticks, colored masking tape, glue sticks, kid’s scissors, crayons, etc.),
- games (i.e., board games, video games), the number of games should be enough to allow each member of each subgroup to participate in the game.

### General (optional)

- Computer to store the data.
- Prizes.
- Camera to take pictures.

## 3. Procedure

### Prior to the event

1. phone or email all event information to parents or guardians,
2. email consent forms and list with recommendations for the day of the event,
3. hold information sessions with all the assistants,
4. completing criminal background checks for all the assistants,
5. training workshops for all assistants.

### Set up (day before)

1. connect all computers and EEG systems,
2. distribute materials in each room as needed,
3. review signed consent forms (email) to make sure all were completed correctly.

### Day of the event

1. entry meeting with all participants and parents or guardians to present the agenda of the day,
2. participants are assigned into their different groups,
3. research team members direct the participants to every room throughout the day until they have completed all activities.

### Example group 1

- station 1: EEG (45 min.),
- break (15 min.),
- station 2: Fun activity,
- break (15 min.),
- station 3: psychometrics (45 min.),
- break and lunch time (60 min.),
- station 4: Fun activity,
- break (15 min.),
- station 5: psychometrics (45 min.),
- break (15 min.),
- station 6: behavioral experiment (45 min.).

For specific information on the EEG station protocol, please see supplementary material (Table 2 in S3 File). Parallel to the children’s activity, parents are welcome to stay and complete parents’ questionnaires (if any) and to participate in workshops offered for them.

### Conclusion

Exit meeting with all participants and parents or guardians to offer thanks and to present a summary of the day’s event showing a slideshow or the kids enjoying the day’s activities and to give each of them gifts.

We performed our study was following the recommendations of the human research ethics guidelines from the Simon Fraser University (SFU) Office of Research Ethics. Written informed consent in accordance with the Declaration of Helsinki was obtained from each parent or guardian, and informed assent was obtained for each participant. The protocol was approved by the office of research ethics at SFU.

The protocol described in this peer-reviewed article is published on protocols.io, dx.doi.org/10.17504/protocols.io.81wgby3y3vpk/v1 and is included for printing as supporting information file 1 with this article.

## 4. Troubleshooting

When considering a camp setting for child data collection, there are two main types of issues that should be considered: issues with the equipment and behavioral issues with participants.

## 5. Anticipated results

In contrast to traditional individual behavioral and neurophysiological data collection settings, the science camp setting allows for the collection of multiple high-quality EEG recordings from children in a single day of testing, as well as the corresponding behavioral assessments. In summary, this protocol offers researchers the tools to create an environment that promotes the optimization of resources (i.e., time, money, data collection), thus helping to collect a greater amount of high-quality data which translates to more representative samples and stronger statistical power. In addition, the science camp provides a framework that could be adapted and replicated for different purposes, populations, and research needs.

## Supporting information

S1 File Step-by-step protocol. (PDF)

S2 File Example of the ad used for recruitment. (DOCX)

S3 File Protocol for group EEG data collection. (DOCX)
