# Supplementary material for: A new acquisition protocol for conducting studies with children: The science camp research experience
Source: PLoS One. 2023 Aug 9;18(8):e0289299. doi: 10.1371/journal.pone.0289299 (PMC10411783; doi:10.1371/journal.pone.0289299)
Supplement: S1 File — (PDF) [file pone.0289299.s001.pdf]

JUL 28, 2023

A NEW ACQUISITION PROTOCOL FOR CONDUCTING STUDIES WITH CHILDREN: THE SCIENCE CAMP RESEARCH EXPERIENCE

Amparo V. Marquez-Garcia[1], Sam M. Doesburg[1], Grace Iarocci[1], Justine R. Magnuson[1], Sylvain Moreno[1]

[1]Simon Fraser University.

Amparo V. Marquez-Garcia

OPEN ACCESS

DOI: dx.doi.org/10.17504/protocols.io.81wgby3y3vpk/v1

Protocol Citation: Amparo V. Marquez-Garcia, Sam M. Doesburg, Grace Iarocci, Justine R. Magnuson, Sylvain Moreno 2023. A NEW ACQUISITION PROTOCOL FOR CONDUCTING STUDIES WITH CHILDREN: THE SCIENCE CAMP RESEARCH EXPERIENCE. **protocols.io** https://dx.doi.org/10.17504/protocols.io.81wgby3y3vpk/v1

License: This is an open access protocol distributed under the terms of the Creative Commons Attribution License, which permits unrestricted use, distribution, and reproduction in any medium, provided the original author and source are credited

Protocol status: Working
We use this protocol and it's working

ABSTRACT

In the last 50 years, the study of brain development has brought major discoveries to education and medicine, changing the lives of millions of children and families. However, collecting behavioral and neurophysiological data from children has specific challenges, such as high rates of data loss and participant dropout. We have developed a science camp method to collect data from children using the benefits of positive peer interactions and interactive and engaging activities, to allow researchers to better collect data repeatedly and reliably from groups of children. A key advantage of this approach is that by increasing participant engagement, attention is also increased, thereby increasing data quality, reducing data loss, and lowering attrition rates. This protocol describes the step-by-step procedure for facilitation of a science camp, including behavioral, electrophysiological, and participatory engagement activities. As this method is robust but also flexible, we anticipate that it can also be applied to different demographics and research needs.

Created: May 03, 2023

Last Modified: Jul 28, 2023

PROTOCOL integer ID: 81357

GUIDELINES

The purpose of this article is to describe the implementation and execution of a science camp for behavioural and electrophysiological data collection in child populations. We recommend that this protocol be implemented by an experienced team in the collection of the selected type of data (i.e., specific behavioral tests) or the use of specific required equipment. We also assume a thorough understanding of the principles of behavioural and cognitive neuroscience. We focus on describing the unique opportunities and challenges presented in group data collection with children. While designing and performing these studies, it is of paramount importance to prioritize participant safety and comfort over research goals, as subjects who consent to participate without any expectation of direct benefit.

Each participant should be evaluated regarding their suitability for the study prior to the day of data collection. Once the study's guidelines have been determined and approved by the relevant ethics review board, we recommend adhering to the following steps involving communication with parents or guardians.As a general recommendation, the participants' parents/caretakers should receive all the information about the testing procedure, including what to expect, how long it will take, etc. We recommend a parent meeting with the research team. It is crucial to ensure that the parent feels that their child will be able to tolerate the experiment without responding negatively to the procedures. Parents should be pre-consulted about the testing session's date, time, and location. If parents and children agree to have their child participate, a simple to-do list should be provided to inform them of all the testing day recommendations. This to-do list is given by parents to children.

All study participants must be enrolled voluntarily, and caregivers and participants must provide informed written consent. In addition, participants must provide assent before each activity, they can decide not to participate in specific activities, and all subjects should be free to withdraw from the study or any activity involved at any time, including during the recording.We have different ways to reach out to our participants: a database and different communication tools: social media and daily newspapers add. See supplementary materials for an example of one of our advertisements used for recruitment.

MATERIALS

The following section provides a detailed illustration of our method providing a concrete example of the broader approach detailed above.

Informed assent from children and informed consent from parents and legal guardians must be obtained.The protocol must be in compliance with appropriate national laws and institutional regulatory board guidelines, and an appropriate ethics review board must approve the protocol for the sessions.

EQUIPMENT

Psychometrics applications and cognitive tests:

Complete psychometric battery and cognitive tests including the specific materials required for each one (i.e., parents' questionnaires, application forms, white paper, colors, pencils, chronometer, etc.). The number of supplies should be enough to record each member of each subgroup of participants.

The electrophysiological recording requires:

- EEG equipment: the number of EEG systems should be enough to record each member of each subgroup of participants,
- cleaning supplies and recording supplies,
- one computer per EEG system with task presentation software,
- one computer per EEG system for data acquisition,
- data analysis software: MATLAB or other similar software is required to analyse the data,
- toys/games: a variety of toys are used to help participants develop rapport with the researchers while they wait for the equipment to be set up. Electric toys are not recommended, instead, sensory toys are recommended due to self-regulatory effects.

Fun stations (examples):

·materials to create crafts (i.e., playdough, paper, watercolor paint, pipe cleaners, craft sticks, colored masking tape, glue sticks, kid's scissors, crayons, etc.),
·games (i.e., board games, video games), the number of games should be enough to allow each member of each subgroup to participate in the game.

General (optional):

·Computer to store the data.
·Prizes.
·Camera to take pictures.

BEFORE START INSTRUCTIONS

Planning

1 Research Team

The large number of participants' data typically collected using this method requires a large group of researchers. The group of assistants could be academics, clinicians, clinical staff, community professionals, and/or students. All should follow

a proper experimental training to conduct the tasks of the protocol and understand how to work with children (including clinical child populations if necessary). All must have completed criminal background checks prior to data collection. Once the background checks have been approved, assistants should sign an agreement of collaboration and participate in the specific training sessions offered by the research group. The training should include an experimental aspect and a clinical aspect (if needed). The experimental aspect of the training should evaluate the previous knowledge of the research team members and create a plan to provide the knowledge they are missing.

After the training, all research team members should be prepared for the activity they were assigned. The group assigned to the administration of psychometric tests should complete the required training for each test (i.e., training varies greatly depending on the test). The groups assigned to a behavioral test that requires the use of a computer and electrophysiological measurement system, or any other equipment designed to make physiological recordings from participants should know how to connect and test all equipment; how to properly manipulate and clean them; how to run the task and record the data.In addition, they should know what to do if the participants refuse to participate in the activity (i.e., What room to direct the participants, what activities to offer instead, etc.).

2 Piloting

Several pilot testing sessions might be needed until all the assistants are capable of mastering the procedures and can do so in a specific timeframe. In the first pilot session, it is recommended that research team members take turns role-playing as participants, as children are not present at this stage. During the clinical part of the pilot stage, general and specific aspects of working with children should be included. Clear rules and expected behavior should always be stated from the beginning.

All research team members should know the characteristics of the group that they will work with and be prepared to act according to the participant's needs, triggers, and level of functioning. One strategy to support this learning phase is to present case studies that represent different situations that teach assistants how to respond to each one. The pilot stage should be repeated as much as needed by the research team members.

3 One Day Prior

In order to ensure a successful group data collection, it is important to set up all data collection stations and run the experiments at least once on the day before the experiment, allowing the research group enough time to solve any last-minute issues that could arise. The research team members should receive clear

instructions about who and how to contact for help if specific problems arise. Necessary checklists and information sheets should be provided for each research team member.

4 Day of the Data Collection

The day starts with an entry meeting in which the children and their parents are welcomed. All the parents sit down and receive a presentation (i.e., using pictures and videos) with the description of each activity and how the science camp works. Children are grouped within their age range. The research team provides children with their name tags and working materials such as t-shirts with different colors per group. The groups are gathered, and the parents and the children are introduced to their group leader (i.e., a research team member in charge of that group) and to their group mates. Time should be allotted to answer questions from parents and children. After introductions, children receive a card to help them collect one sticker for each activity completed. Research team members then lead their groups through the circuit of the science camp. Each group leader will stay the whole day with their group. It provides stability and reassurance for the children.Parents are welcome to stay in the registration room and participate in workshops, or they can run errands and come back before the exit meeting.At the end of the day, the protocol ends with an exit meeting. During this meeting, all groups of children, research team members and parents are reunited for a debriefing and answer any questions they may have. A recap of the event (i.e., showing pictures of the event to the parents) and prizes are provided. The research team will also inform the parents when they will be able to receive a report of the day.No individual report is shared with participant information but a global day description of what the children have done during the science camp protocol explaining the goal of the studies is encouraged to create a long-term relationship with the parents and their child.

4.1 Data Acquisition

To achieve a science camp protocol, we carefully planned a step-by-step one-day agenda. Different rooms are conditioned for different activities (see example in Fig 1). During the camp (approximately 9:00 a.m. to 5:00 p.m.), participants visit each of the stations where they will perform research-related and non-research related activities.Depending on the research question and the characteristics of the participants' population, the activities, set up, and duration should be adapted.They will receive breaks in between each activity to be able to rest and engage with other participants (see example in Fig 2). In addition to the research and fun stations, it is necessary to prepare a room big enough to hold meetings with all the parents and participants.Fun station are two extra rooms for breaks, in these rooms there is always a research team member prepared to receive any participants who need a break. One of them is called the calm room; this room is always quiet. The second

room is called the playroom; in this room, participants can take a break from the planned activities, and they have some extra options for fun activities to participate in (i.e., play board games, sing, draw or work on crafts).

Figure 1: Science camp layout example.

Figure 1: Science camp layout example.

Figure 2. Science camp agenda showing the distribution of the groups and the activities.

In summary, the implementation of a science camp protocol will typically use at least three different types of stations. The first is for the behavioural evaluations; the second is the 'fun' stations, and the third is the electrophysiological station.

4.2 Science camp behavioral assessment

The configuration of the rooms allows us to have the required privacy for psychometrics application or behavioural assessment simultaneously. It is important to have separate workstations with light divisions (i.e., Wood or plastic panels) to prevent children from interfering with each other's activities in the same room (please see example in Fig 3).

Figure 3. Example of layout in the room for psychometrics application.

Figure 3. Example of layout in the room for psychometrics application.

4.3 Science camp electrophysiology recordings

For the EEG station, our research group uses 5 EEG systems (i.e., the number of systems will depend on the space possibilities). The EEG station should be located in a room where EEG systems can be at least 3 meters apart and have one cleaning station in the center of the room (See Fig 4). Our data verification tests showed that 3 meters was enough to effectively minimize the signal contamination between systems. Having the cleaning station in the center of the room is convenient

towards preparation of the stations between groups.

After the accent of the participants, they are guided to their station (one per participant). Two research assistants manage each station (i.e., two research assistants per station and participant). Within the first 20 minutes, the two research assistants explain the tasks' instructions to each participant and prepare the settings (i.e., capping the participants and training them on the task).

In this setup, an essential factor is the orientation of the participants (i.e., not seeing each other), as this will limit the distraction and subsequent loss of data. During this time (the first 20 minutes), participants are also allowed to talk with the research assistants and move in their seats. For some participants, research assistants could also propose watching a short children's movie to the participants.

The total length of the EEG recording depends on the research questions, task design and population. Previous tasks with children for our research group lasted up to 30 minutes, providing breaks between experimental blocks to allow them to stretch and move in their seats. Between groups, 15 minutes are necessary to clean and prepare the settings for the next group.

In total, 13 research assistants are needed in this station example. Each EEG system is operated by 2 research assistants. There is also one assistant coordinating the groups' transitions, overseeing the overall data collection, and providing extra support if needed, another assistant ready to provide technical support and one more assistant working exclusively in the cleaning station preparing the supplies for all the groups.

Figure 4. Layout of EEG recording room.

Figure 4. Layout of EEG recording room.

4.4 Science camp fun stations

These rooms can have a flexible configuration, but they should be safe for child play. These stations are where the participants have fun and interact with each other. For example, they can play board games, sing, draw or work on crafts. These stations can be adapted to the space and to the activities available and best suited to the participant populations. There is no need for a special configuration or separation between participants.

1 Prior to the event:

1. phone or email all event information to parents or guardians,
2. email consent forms and list with recommendations for the day of the event,
3. hold information sessions with all the assistants,
4. completing criminal background checks for all the assistants,
5. training workshops for all assistants.

2 Set up (day before):

1. connect all computers and EEG systems,
2. distribute materials in each room as needed,
3. review signed consent forms (email) to make sure all were completed correctly.

3 Day of the event:

1. entry meeting with all participants and parents or guardians to present the agenda of the day,
2. participants are assigned into their different groups,
3. research team members direct the participants to every room throughout the day until they have completed all activities.

Example group 1:
a.station 1: EEG (45 min.),
b.break (15 min.),
c.station 2:Fun activity,
d.break (15 min.),
e.station 3: psychometrics (45 min.),
f.break and lunch time (60 min.),
g.station 4:Fun activity,
h.break (15 min.),
i.station 5: psychometrics (45 min.),
j.break (15 min.),
k.station 6: behavioral experiment (45 min.).

4 For specific information on the EEG station protocol, please see supplementary material.Parallel to the children's activity, parents are welcome to stay and complete parents' questionnaires (if any) and to participate in workshops offered for them.

Conclusion:
Exit meeting with all participants and parents or guardians to offer thanks and to present a summary of the day's event showing a slideshow or the kids enjoying the day's activities and to give each of them gifts.
