# Supplementary figures and images for: A new acquisition protocol for conducting studies with children: The science camp research experience
Source: PLoS One. 2023 Aug 9;18(8):e0289299. doi: 10.1371/journal.pone.0289299 (PMC10411783; doi:10.1371/journal.pone.0289299)

S2. Supplementary material 2.

Fig 5. Example of the ad used for recruitment.

**
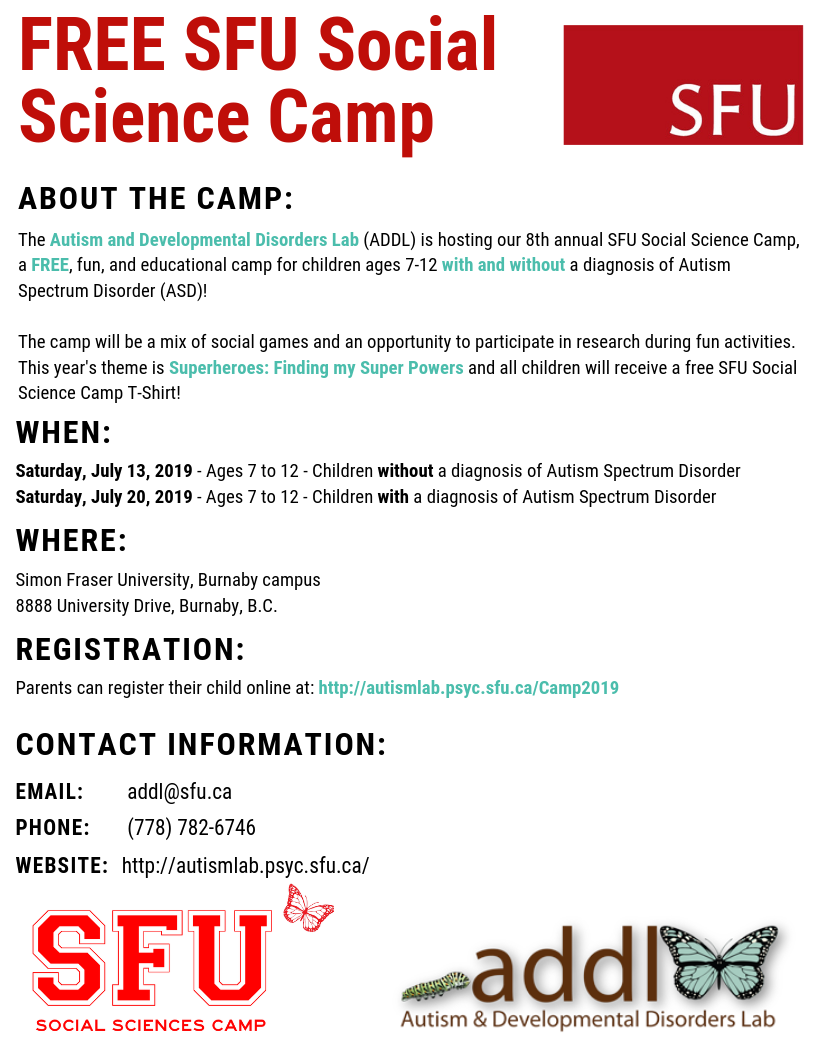
**

Supplement: S2 File — (DOCX) [file pone.0289299.s002.docx]
