# Supplementary material for: A new acquisition protocol for conducting studies with children: The science camp research experience
Source: PLoS One. 2023 Aug 9;18(8):e0289299. doi: 10.1371/journal.pone.0289299 (PMC10411783; doi:10.1371/journal.pone.0289299)
Supplement: S3 File — (DOCX) [file pone.0289299.s003.docx]

S3. Supplementary material 3:

Table 2. Protocol for group EEG data collection.

1. Upon arrival to the EEG station *5 minutes

- Ask participants if they need to use the restroom.
- Send each participant to each one of the tables with the equipment.
- Ask participants to remove eyeglasses, hair clips, & earrings.
- If a participant has long hair, ask to tie it up in a low ponytail or sweep behind their ears.
- Let the participant feel the syringe and put some gel on their fingers so they can understand that it is not dangerous (not sharp, hot, etc.)
- Start playing a movie for participants or give them something to keep them occupied.
- Ask for permission to touch the participant prior to performing each of the following steps. Do not startle them at any point during the protocol (i.e. adding gel to an electrode location without their permission).
- Explain the experiment to the participant in detail (using a custom written script).

*Note: The following is an example of the protocol that we follow in our lab with our equipment, but these steps can vary depending on the equipment you are using.*

2. Applying the EEG cap. *15 minutes

- Gently wipe the skin surface directly above the left eyebrow and beside the left eye (~1cm from the corner of the eye between the eye and the ear) with an alcohol wipe.
- Ask the participant to look straight ahead and apply the vertical EOG electrode directly above the pupil on the forehead, as close to the eyebrow as possible without attaching to any hair. Place the horizontal EOG electrode approximately 1cm from the corner of the eye.
- Move EOG electrode wires so that they are directed backwards, toward the participant’s ear.
- Gently wipe the participant’s earlobe or mastoids (whichever location will be used for the reference electrode).
- Measure the distance between the union (back of head) to the nasion (between eyebrows) and between both preauricular points.
- Place cap on participant’s head so that the Cz electrode location is centered at the intersection between the inion and nasion, and the preauricular points.  The FPz electrode should be 10% of the distance between the nasion and the inion, measured from the nasion.
- Fasten the chin strap. Ensure that the participant is comfortable & can talk and swallow without difficulty. Put a gauze pad under the chin strap if it is irritating to the participant.
- Take the plastic syringes and fill them up with electrode gel and begin inserting the gel into each of the labeled electrode sites.  Use the weight of the syringe when adding gel to the electrodes (you do not need to push down on the syringe). Insert the gel slowly into the electrode site releasing gel as you move the syringe out of the electrode location (wiggling it slightly).  Use a wooden dowel to lightly abrade, move hair out of the way, and check for air pockets. Add more gel if necessary.
- Attach electrode cables to the appropriate locations, including the EOG, CMS/DRL, and EEG electrode positions.
- Let all electrode cables fall neatly at the back of the participant so that they cannot be touched or moved during the recording, in order to minimize noise in the signal.  Tape them to the back of the participant’s shirt if necessary.
- If necessary, carefully place participant’s glasses back on.

3. Acquisition/Presentation computer setup: *5 minutes + time to complete task

- Follow step-by-step procedures for properly setting up EEG acquisition devices (see example attached for Enobio’s NIC system).
- Ensure that impedance values are below ~20 kΩ for EOG and EEG channels.  Add more gel if necessary to bring these values down.
- Allow participants to practice the task for a sufficient period of time (depending on the task/learning effect)
- Run the task presentation (see example attached for running file using Presentation software).

4. After the experiment: *5 minutes

- Remove the cap and the electrodes from the participant’s head.
- Thank the participants and offer a small reward.
- Walk the participants to their group leader so they can go to the next station.
- Clean equipment and prepare it for the next group of children.

5. Receive the next group of children and repeat all the data collection steps.

6. The children who participated in the EEG data collection station proceed to participate in the next station’s activities (see Fig. 2 and Fig. 3).
